# Introduction: Validation methods for function genome annotation

**DOI:** 10.1186/1471-2164-12-S1-I1

**Published:** 2011-06-15

**Authors:** Marvin Stodolsky

**Affiliations:** 1 Retired from the US Department of Energy, DOE Office of Biological and Environmental Research, 1991-2010

## 

This supplement comprises a report on progress during 2007-10 on an outstanding and continuing problem. What are effective and high throughput methodologies for validating the function of genes, newly displayed within DNA sequences? The display of genomes as DNA sequence is becoming increasingly cheap and fast. Sequence comparison methodologies do serve for computationally recognizing homologues between genes in a newly displayed genome, with those of previously analyzed genomes. This is a first automated step in annotation of newly recognized, candidate genes. But there are abundant examples that follow through experimentation is needed to improve and often also to correct the first outputs of computational annotation. For newly displayed microbial genomes, about a third of the genes do not thus acquire any functional assignment. For the more complex genomes of higher species, gene coverage by automated annotation is even poorer.

The DOE Office of Biological and Environmental Research (BER) participated in a 2006 workshop sponsored by the NIH National Center for Biological Information (NCBI). Numerous limitations of computational annotation were discussed. DOE responded with a call for research applications in May 2007: http://www.sc.doe.gov/grants/FAPN07-14.html . Completely solving the long standing problem of validating annotation was certainly not feasible. Rather BER sought methods which could be applied in high throughput modes, with particularly applicability to gene families or genomes related to DOE missions. Validation projects initiated through the FAPN07-14 call and review process are included herein, with complementation by a few cogent projects started earlier.

Validation of gene function certainly remains an outstanding problem arena. But the suite of accomplishments and methods reported herein will surely be useful to those considering validation battles.

